# Predicting the requirement for renal replacement therapy in intensive care patients with sepsis

**DOI:** 10.1186/s13054-018-2135-5

**Published:** 2018-08-20

**Authors:** Axel Nierhaus, Frank Bloos, Darius Cameron Wilson, Gunnar Elke, Patrick Meybohm, the SepNet Critical Care Trials Group

**Affiliations:** 1 Department of Intensive Care Medicine, University Hospital Hamburg-Eppendorf, Hamburg, Germany; 2 Department of Anesthesiology and Intensive Care Medicine, Jena University Hospital, Am Klinikum 1, 07747 Jena, Germany; 3 Center for Sepsis Control & Care (CSCC), Jena University Hospital, Am Klinikum 1, 07747 Jena, Germany; 4 B·R·A·H·M·S GmbH, Hennigsdorf, Neuendorfstr. 25, 16761 Hennigsdorf, Germany; 5 Department of Anaesthesiology and Intensive Care Medicine, University Medical Center Schleswig-Holstein, Campus Kiel, Arnold-Heller-Str. 3 Haus 12, 24105 Kiel, Germany; 6 Department of Anaesthesiology, Intensive Care Medicine and Pain Therapy, University Hospital Frankfurt, Theodor-Stern-Kai 7, 60590 Frankfurt am Main, Germany

Sepsis is one of the most frequent causes of acute kidney injury (AKI) in critically ill patients, with initial organ impairment often followed by dysfunction in other systems [1]. Renal dysfunction may therefore represent one facet in the evolution towards multiple organ dysfunction syndrome (MODS) or, alternatively, may be indicative of system-wide endothelial damage caused by hyperinflammation and a positive fluid balance. Whilst numerous biomarkers have been investigated to predict renal replacement therapy (RRT) requirement, including NGAL, TIMP-2 and IGFBP-7 [2], mid-regional proadrenomedullin (MR-proADM) may also be of interest due to its involvement in capillary leakage, endothelial dysfunction and the initial stages of multiple organ failure development [3, 4].

In a secondary analysis of 1089 severe sepsis and septic shock patients enrolled in the SISPCT trial [5], RRT was initiated in 322 (29.9%) patients within the first 21 days of treatment, including 178 (55.5%; 52.2% mortality) patients at baseline and 118 (36.6%; 55.1% mortality) additional patients between days 1-7. Continuous veno-venous haemodialysis (CVVHD: *N* = 88; 49.4%) and haemodiafiltration (CVVHDF: *N* = 54; 30.3%) were the most common modes of RRT at baseline.

Biomarker (PCT, MR-proADM, CRP and lactate) and standard clinical and laboratory parameters (creatinine, urea and 24-h urine output) were subsequently compared to identify RRT requirement at baseline (day 0), and predict requirement between days 1 and 7 in patients where no RRT was previously initiated. AUROC and logistic regression analysis found that urine output, MR-proADM and creatinine performed similarly in identifying RRT requirement at baseline, whereas MR-proADM more accurately predicted requirement between days 1 and 7 (Fig. 1). Previously established [3] MR-proADM cut-offs for predicting 28-day mortality found that increasing (e.g. moderate to high: *N* = 19; 47.5%; OR [95% CI]: 67.6 [18.5 - 247.2]) or continuously elevated (*N* = 35; 64.8%; OR [95% CI]: 137.5 [38.7 - 489.1]) concentrations over the first 24 h in patients where no RRT was initiated at baseline resulted in a high likelihood of subsequent RRT requirement. Conversely, few cases of RRT over the first 21 days of ICU therapy were initiated in patients with continuously low (*N* = 3; 1.3%) or decreasing (moderate to low: *N* = 1; 1.3%) MR-proADM concentrations.

**Fig. 1:**
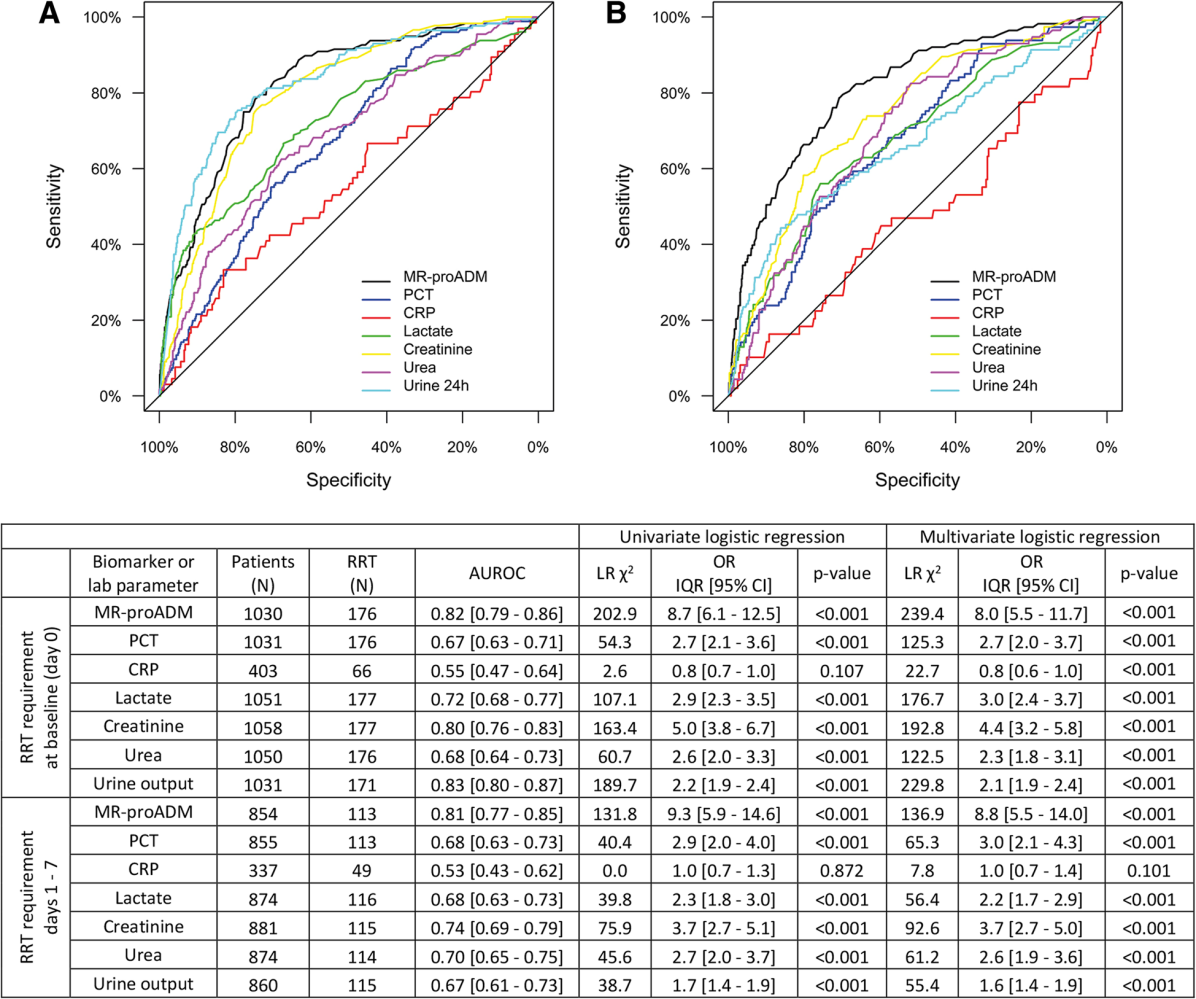
Identification of patients requiring renal replacement therapy (RRT) upon sepsis diagnosis and over the first week of ICU treatment. Logistic regression and AUROC analysis for the requirement of RRT in all patients at baseline (**a**) and during the first 7 days of ICU therapy in patients with no prior RRT (**b**). Multivariate Cox regression analysis was corrected for age and the presence of comorbidities. *AUROC* area under the receiver operating characteristic curve, *CI* confidence interval, *CRP* C-reactive protein, *IQR* interquartile range, *LR* likelihood ratio, *MR-proADM* mid-regional proadrenomedullin, *N* number, *OR* odds ratio, *PCT* procalcitonin, *RRT* renal replacement therapy

Results suggest that increasing or continuously elevated MR-proADM concentrations, indicative of increased capillary leak, may be a useful predictor of RRT requirement during ICU therapy. Further studies are required to investigate the relationship between MR-proADM, positive fluid balance and renal replacement therapy in critically ill patients with sepsis.

## Abbreviations

AKI: Acute kidney injury
AUROC: Area under the receiver operating characteristic curve
CI: Confidence interval
CRP: C-reactive protein
CVVHD: Continuous veno-venous haemodialysis
CVVHDF: Continuous veno-venous haemodiafiltration
ICU: Intensive care unit
IGFBP-7: Insulin-like growth factor-binding protein 7
MODS: Multiple organ dysfunction syndrome
MR-proADM: Mid-regional proadrenomedullin
N: Number
NGAL: Neutrophil gelatinase-associated lipocalin
OR: Odds ratio
PCT: Procalcitonin
RRT: Renal replacement therapy
SISPCT: Placebo-controlled trial of sodium selenite and procalcitonin guided antimicrobial therapy in severe sepsis
TIMP-2: Tissue inhibitor of metalloproteinases 2
